# Post-appendectomy fibrocalcific lesion at the appendiceal orifice mimicking a gastrointestinal stromal tumor

**DOI:** 10.1055/a-2876-1818

**Published:** 2026-06-01

**Authors:** Bin Deng, Chao Sun, Mengxian Ju, Jun Liu, Keshi Yan, Weizhao Wang

**Affiliations:** 1 Department of Endoscopy Center 370089 Northern Jiangsu People’s Hospital Affiliated to Yangzhou UniversityYangzhouChina; 2 Department of Endocrinology 370089 Northern Jiangsu People’s Hospital Affiliated to Yangzhou UniversityYangzhouChina; 3 Department of Anesthesiology 370089 Northern Jiangsu People’s Hospital Affiliated to Yangzhou UniversityYangzhouChina

**Fig. 1:**
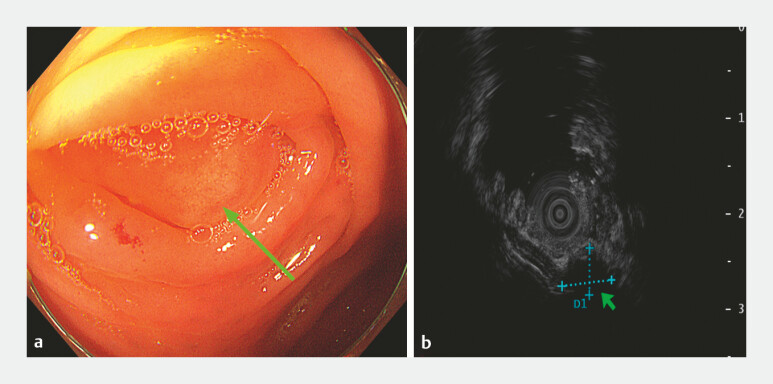
Endoscopic and endosonographic appearance of the lesion (green arrow). **a** White-light colonoscopy showing a subepithelial-appearing lesion at the appendiceal orifice. **b** Endoscopic ultrasound showing a 7 × 7 mm hypoechoic lesion, apparently arising from the muscularis propria.

A 25-year-old man was referred after routine colonoscopy revealed a subepithelial-appearing lesion at the appendiceal orifice ( Fig. 1 **a** ). He had undergone appendectomy 9 years earlier and had no gastrointestinal symptoms. Physical examination and laboratory tests, including tumor markers, were unremarkable.

**Fig. 2:**
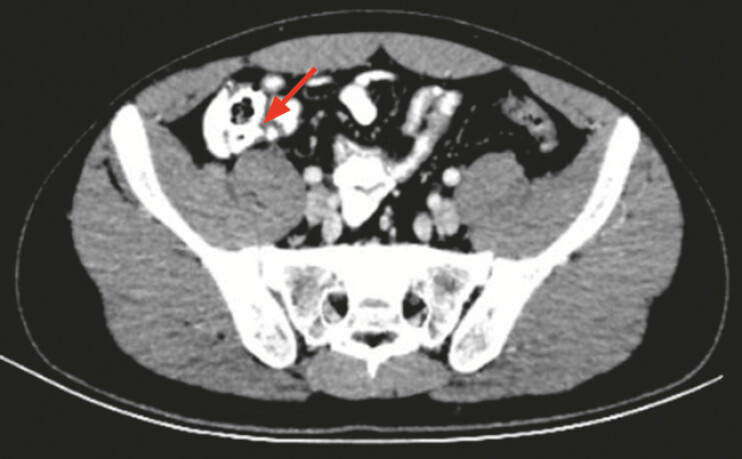
Contrast-enhanced computed tomography showed mild focal thickening of the ileocecal wall without a definite mass (red arrow).

Endoscopic ultrasound demonstrated a 7 × 7mm hypoechoic lesion at the appendiceal orifice, apparently arising from the muscularis propria ( Fig. 1 **b** ). Contrast-enhanced computed tomography showed mild focal thickening of the ileocecal wall without a definite mass ( Fig. 2 ), raising suspicion for a gastrointestinal stromal tumor. Because appendiceal-orifice lesions may be difficult to characterize and can mimic true subepithelial tumors, endoscopic resection was undertaken ( Video 1 ) for diagnosis and treatment 1 2 .

Endoscopic submucosal dissection of a post-appendectomy fibrocalcific lesion at the appendiceal orifice mimicking a gastrointestinal stromal tumor, followed by clip closure of the resection defect.Video 1

**Fig. 3:**
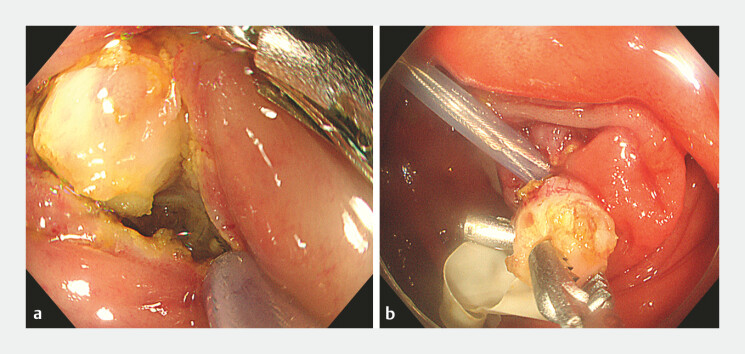
Endoscopic submucosal dissection of the appendiceal-orifice lesion. **a** Mucosal incision over the lesion, and the lesion clearly exposed after stepwise submucosal dissection. **b** En bloc resection completed using a snare with rat-tooth forceps assistance.

**Fig. 4:**
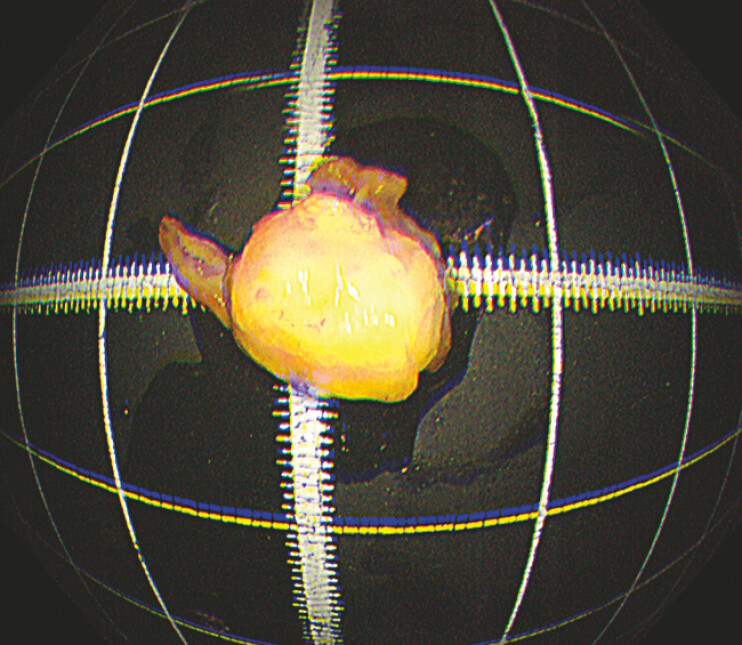
Complete resection of the lesion, measuring approximately 7 × 7 mm.

After mucosal incision, the lesion was clearly exposed and dissected stepwise under direct visualization ( Fig. 3 **a** ). En bloc resection was completed using a snare with rat-tooth forceps assistance ( Fig. 3 **b** , Fig. 4 ). A minor muscularis injury occurred during resection and was immediately closed with hemoclips. The patient developed transient postoperative fever (39.0℃), which resolved after anti-infective therapy. He was discharged uneventfully.

**Fig. 5:**
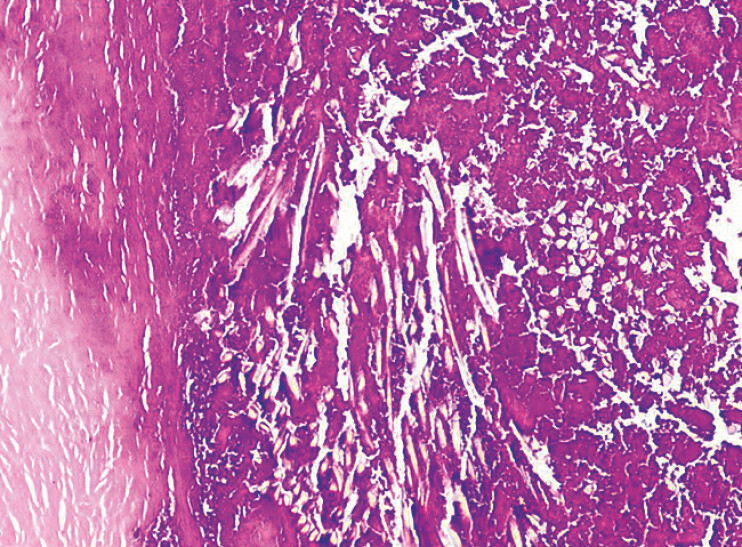
Hematoxylin and eosin staining showing fibrocollagenous hyperplasia with focal calcific deposition, and no tumours or malignant lesions were observed.

Histopathology showed fibrocollagenous hyperplasia with focal calcific deposition, without stromal tumor or malignancy ( Fig. 5 ). Previous reports have shown that post-appendectomy stump-related lesions, including appendiceal inversion and other late postoperative changes, may present as cecal mass-like lesions and mimic neoplasia 3 4 5 . Our case expands this spectrum by showing that a benign fibrocalcific degenerative lesion may mimic a muscularis propria-derived tumor on endoscopic ultrasound. In selected patients, endoscopic submucosal dissection can provide definitive diagnosis and minimally invasive treatment while avoiding unnecessary surgery 1 .

Endoscopy_UCTN_Code_CCL_1AD_2AI
